# Processing Suitability of Physical Modified Non-GMO High-Amylose Wheat Flour as a Resistant Starch Ingredient in Cookies

**DOI:** 10.3390/molecules30122619

**Published:** 2025-06-17

**Authors:** Yujin Moon, Meera Kweon

**Affiliations:** 1 Department of Food Science and Nutrition, Pusan National University, Busan 46241, Republic of Korea; amoebacul@pusan.ac.kr; 2 Kimchi Research Institute, Pusan National University, Busan 46241, Republic of Korea

**Keywords:** high-amylose wheat flour, physical treatment, heat-moisture treatment, cookie processing, digestibility

## Abstract

High-amylose wheat (HAW), developed through non-genetic modification, addresses the growing demand for clean-label and nutritionally enhanced food products. This study systematically investigated the effects of heat-moisture treatment (HMT; 20% and 25% moisture levels) on the physicochemical properties and cookie-making performance of HAW flour (HAWF) and soft wheat flour (SWF). HMT promoted moisture-induced agglomeration, leading to increased particle size, reduced damaged starch content, and enhanced water and sucrose solvent retention capacities. Although the amylose content remained largely unchanged, pasting behavior was differentially affected, with increased viscosities in SWF and slight decreases in HAWF. Thermal analyses demonstrated elevated gelatinization temperatures, indicating improved thermal stability, while X-ray diffraction revealed alterations in starch crystallinity. Furthermore, HMT weakened gluten strength and modified dough rheology, effects more pronounced in HAWF. Cookies prepared from HMT-treated flours exhibited larger diameters, greater spread ratios, and reduced heights. In vitro digestibility assays showed a marked reduction in rapidly digestible starch and increases in slowly digestible and resistant starch fractions, particularly in HAWF cookies. Collectively, these findings establish HMT as an effective strategy for modulating flour functionality and enhancing cookie quality, while concurrently improving the nutritional profile through the alteration of starch digestibility characteristics.

## 1. Introduction

Wheat is one of the most widely consumed cereals globally, and it serves as a key ingredient in various products, including bread, pasta, and cookies. In Korea, annual wheat consumption is approximately 2 million tons, driven by the increased adoption of Western dietary habits [1]. Additionally, growing consumer awareness of health and nutrition has shifted the focus of the food industry toward the development of functional or nutraceutical foods. This trend is particularly evident in Korea, where traditional rice-based diets are increasingly being replaced by wheat-based products with added health benefits.

Resistant starch (RS), which resists digestion in the small intestine [2], has emerged as a key functional ingredient because it enhances gut health, regulates blood glucose levels by reducing postprandial glucose spikes, and acts as a prebiotic dietary fiber by increasing short-chain fatty acid production in the colon [3,4,5]. These benefits position RS as a valuable starch component for reducing the risk of chronic conditions, such as diabetes and obesity, which are growing public health concerns worldwide. Among the five types of RS, Type II (native high-amylose starch) and Type III (retrograded starch) are produced by physical treatment. As consumer interest in natural and clean-label ingredients grows, heat-moisture treatment (HMT) of starch, a physical starch modification without using chemical additives, has gained attention for enhancing the RS content [6].

HMT, performed at moisture levels below 30% (*w*/*w*), enhances starch crystallinity and reduces digestibility through controlled heating at temperatures between the glass transition (Tg) and gelatinization (Tgel) points [7]. At the molecular level, HMT rearranges amylose and amylopectin chains, strengthening hydrogen bonds and forming a more ordered structure, which impedes enzymatic access [8]. This effect is more pronounced in high-amylose starches, which yield higher RS levels than regular starches [9]. However, the outcomes depend on factors such as the moisture content, temperature, and treatment duration, which require optimization for specific applications [10,11].

High-amylose wheat flour (HAWF) has gained attention as a functional RS-containing ingredient owing to its high amylose-to-amylopectin ratio, which slows starch digestion and provides metabolic benefits, such as prolonged satiety and improved glucose regulation [12,13]. Unlike genetically modified high-amylose corn starch, non-genetically modified (non-GMO) high-amylose wheat, developed through conventional breeding, meets clean-label demands and offers a natural dietary fiber source [14]. Additionally, owing to its elevated amylose content, it exhibits distinct physicochemical properties such as a higher gelatinization temperature and lower swelling power than standard soft wheat flour (SWF), rendering it a promising ingredient for innovative baked goods [15]. Cookies, a popular wheat-based product, provides a practical platform for incorporating HAWF, offering the potential for reduced glycemic response and improved digestive health—benefits that appeal to both health-conscious consumers and food manufacturers. Although HMT has been shown to significantly increase the RS content in high-amylose corn and rice starches, its application to HAWF remains underexplored [9,16]. Currently, most commercial RS products are derived from native or physically modified high-amylose starches or chemically modified starches. When incorporated into wheat-based products such as baked goods or noodles, they dilute the gluten functionality, necessitating the addition of costly active gluten or gluten-enhancing agents. In contrast, HAWF can be used “as is” without negatively affecting gluten functionality, providing a clear advantage. In cookie production, the high sugar content suppresses gluten development during mixing. Moreover, HMT can denature gluten proteins and weaken dough strength—changes that are actually desirable for achieving favorable cookie texture and quality.

We hypothesized that applying HMT to HAWF would enhance cookie-making performance and nutritional benefits by increasing slowly digestible starch (SDS) and RS content, enhancing the suitability of HAWF for cookies that exhibit low glycemic impact and improve gut health. We compared the physicochemical properties and in vitro digestibility of HAWF subjected to HMT at 20% (HMT-20) and 25% (HMT-25) moisture levels with those of commercial SWF by analyzing starch pasting behavior, crystallinity, gelatinization characteristics, and enzymatic digestibility. Additionally, we evaluated the digestibility of cookies prepared from HMT-treated HAWF to assess their nutritional benefits.

## 2. Results and Discussion

### 2.1. Particle Size of Wheat Flours

**Figure 1:**
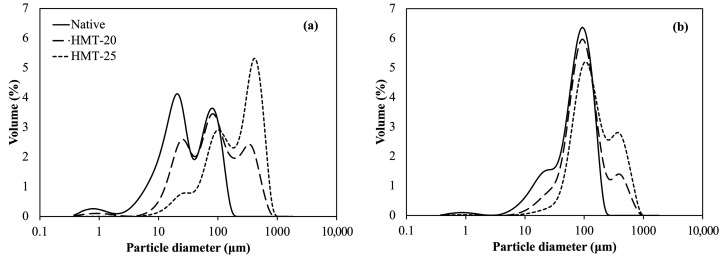
Particle size distribution of wheat flour samples. (**a**) SWF, soft wheat flour; (**b**) HAWF, high-amylose wheat flour.

Figure 1 and Table 1 present the particle size distributions and sizes, respectively, of the flour samples. The d50 values for native, HMT-20, and HMT-25 samples were 28.1, 83.4, and 236.3 μm, respectively, for SWF, and 78.7, 100.5, and 141.8 μm, respectively, for HAWF. The results indicated that HMT increased the average particle size of flour, and this increase was significantly greater with higher moisture levels. Native SWF and HAWF were obtained from different manufacturers, resulting in differences in particle size distribution. Wheat flour particle size is influenced by a range of factors, including wheat cultivar, kernel hardness, moisture content, and milling equipment used [17].

This result can be primarily attributed to the functional components in the flour that are associated with water-holding capacity. Unlike starch alone, damaged starch, arabinoxylans, and gluten proteins are major flour components that contribute to water retention. In HMT, water is added to the flour, and these flour components compete for water absorption, leading to a non-uniform distribution of moisture [18]. Upon subsequent heat treatment, flour particles with higher moisture levels agglomerate more easily, resulting in denser particles and increased particle sizes. Most studies on HMT have focused on cereal starch rather than flour, reporting changes in starch granule surface characteristics and size; however, some of these findings are contradictory [19,20]. Lv et al. [21] reported starch granule aggregation and protein denaturation following HMT of highland barley flour, which aligns with our observations.

### 2.2. Moisture, Amylose and Starch Damage Content of Wheat Flours

Table 2 presents the moisture, amylose, and damaged starch contents of the wheat flour samples. The moisture content of the native SWF and HAWF samples was 13.6% and 15.2%, respectively. In contrast, the HMT samples exhibited reduced moisture contents, ranging from 10.0% to 10.8% for SWF and 10.2% to 11.6% for HAWF, which indicates a significant decrease during the cooling process at 20–25 °C.

The amylose content of the flour samples showed no significant changes after HMT. Du et al. [22] reported an increase in the amylose content of chickpea starch after HMT with adjusted moisture levels of 15–30% at 110 °C for 4 h, attributing the increase to the conversion of amylopectin chains into amylose. In contrast, Kaur et al. [23] observed a decrease in the amylose content of oat starch following HMT (30% moisture, 100 °C for 16 h). Numerous studies have suggested that HMT promotes additional interactions between amylose–amylose and amylose–amylopectin chains, which alter the crystalline structure of starch and decrease amylose solubility, leading to an underestimation of amylose content [24]. Furthermore, the increased formation of lipid complexes with amylose chains contributes to a reduction in the measurable amylose content, indicating that HMT effectively alters the molecular structure of starch. The impact of HMT on amylose content varies depending on the starch source [25].

HMT reduced the damaged starch content across all samples. Specifically, the damaged starch content decreased from 3.4% to 2.0% (HMT-20) and 2.8% (HMT-25) in SWF and from 4.7% to 1.5% (HMT-20) and 1.6% (HMT-25) in HAWF. These findings are consistent with those of Liu et al. [26], who demonstrated that HMT effectively decreases the level of damaged starch in ball-milled starches with deliberately increased damage. This suggests that HMT modifies starch properties by restructuring the disordered regions of damaged starch and enhancing its granule stability.

### 2.3. Solvent Retention Capacity (SRC) of Wheat Flours

Table 2 lists the SRC values of the flour samples. The SRC values for SWF in water, lactic acid, sodium carbonate, and sucrose solutions were 51.2%, 103.1%, 72.0%, and 96.4%, respectively, while those for HAWF were 75.5%, 118.9%, 89.5%, and 128.5%, respectively. The SRC values of native SWF in the four solvents were lower than those of HAWF, indicating a lower water-holding capacity and reduced contribution of gluten protein, damaged starch, and arabinoxylans toward water retention. This characteristic renders SWF more suitable for cookie production, as it facilitates moisture evaporation during baking and contributes to a desirable crisp texture [18]. Cookies typically have a moisture content of approximately 2–3%, and effective moisture loss during baking is essential for maintaining quality during storage. Although the arabinoxylan content in flour is generally less than 1%, it can absorb up to 10 times its weight in water, considerably affecting cookie quality. The sucrose SRC is primarily associated with arabinoxylans (also known as pentosans) in the flour and is considered one of the most important predictors of cookie quality, particularly cookie diameter [27]. A strong negative correlation between the sucrose SRC and cookie diameter has been reported [28,29,30].

Following HMT, a significant increase in water-retention capacity was observed, with a more pronounced effect in HAWF than in SWF. The enhanced water absorption capacity of the flour after HMT may be attributed to the enlarged particle sizes, which resulted in less densely packed pellets after centrifugation, allowing more water to be retained.

In contrast, HMT significantly decreased the lactic acid SRC values (*p* < 0.05), likely because of the denaturation of gluten proteins and the restricted swelling of the protein network. Even dry heat treatment of wheat flour (applied “as is,” without moisture adjustment) has been shown to cause protein aggregation, which hinders gluten development and network formation during dough mixing [31,32]. Additionally, Steertegem et al. [32] reported a reduction in free sulfhydryl groups and protein extractability in a sodium dodecyl sulfate (SDS)-containing medium in wheat flour subjected to dry heat treatment. Sodium carbonate SRC values slightly decreased or increased after HMT, possibly due to the inconsistent effects of HMT on the swelling behavior of heat-damaged starch granules and the enlargement of particle sizes. After HMT, the sucrose SRC values decreased for SWF but increased for HAWF. A consistent trend was reported by Steertegem et al. [33], who observed a decrease in the lactic acid SRC, increases in the water and sucrose SRC values, and a slight decrease in the sodium carbonate SRC in wheat flour treated with dry heat.

In summary, HMT induces structural changes in the starch and protein components of wheat flour, disrupting the granular, crystalline, and helical structures of starch and thereby altering the functional properties of the flour [34]. These effects are particularly evident at high moisture levels, where the interactions between water and the polar groups of carbohydrates enhance water uptake.

### 2.4. Pasting Properties of Wheat Flours

**Figure 2:**
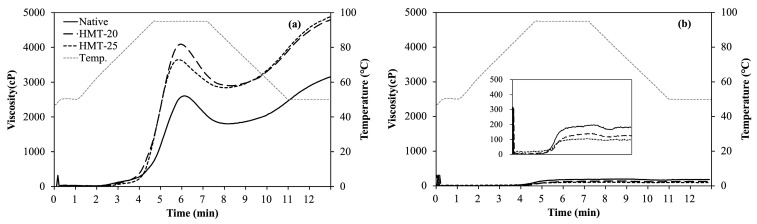
Rapid visco-analyzer (RVA) pasting curves of the wheat flour samples. (**a**) SWF, soft wheat flour; (**b**) HAWF, high-amylose wheat flour.

Figure 2 shows the rapid visco-analyzer (RVA) pasting curves of the flour samples, and Table 3 presents their pasting properties.

SWF samples, including native and HMT, exhibited remarkably higher pasting viscosities (2755–4241 cP) and lower pasting temperatures (64.5–71.0 °C) than HAWF samples (283–368 cP and 81.2–82.3 °C, respectively). The lower pasting viscosities and higher pasting temperatures of HAWF were attributed to the resistant swelling of starch granules with higher amylose content when heated to 95 °C and subsequently exposed to continuous shear, indicating the difficulty of granule disruption [35,36]. Additionally, the presence of amylopectin with long branched chains contributes to an increased pasting temperature [37]. Moon and Kweon [38] reported that the amylopectin structure of starch isolated from HAWF (57.6% amylose content) contained a significantly larger proportion of long branched chains than that of starch isolated from normal wheat flour (28.9% amylose content).

HMT increased the pasting viscosity of SWF, indicating enhanced swelling of starch granules during heating. HMT-20 exhibited a slightly higher peak viscosity than HMT-25. In contrast, HAWF showed the opposite trend: HMT inhibited starch granule swelling, resulting in decreased pasting viscosities for both HMT-20 and HMT-25; however, the overall pasting viscosities remained low. This observation is consistent with the findings of Ambigaipalan et al. [39], who reported that thermal stability was improved and gelatinization inhibited when HMT was applied to pulse starches with a B-type crystalline structure. The effects of HMT on starch pasting properties vary widely, primarily because of differences in starch sources and HMT conditions [8]. Numerous studies [26,40,41] have reported that HMT significantly reduces swelling power and solubility, thereby altering starch-related properties.

### 2.5. Thermal Properties of Wheat Flours

**Figure 3:**
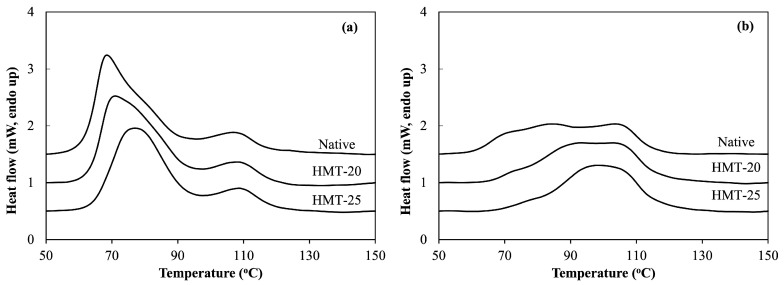
Differential scanning calorimetry (DSC) thermograms for wheat flour samples. (**a**) SWF, soft wheat flour; (**b**) HAWF, high-amylose wheat flour.

Figure 3 presents the differential scanning calorimetry (DSC) thermograms of the flour samples, and Table 4 shows their thermal properties. The first peak corresponds to the gelatinization phase transition of starch granules, whereas the second peak represents the melting of the amylose-lipid complex. Native HAWF exhibited a considerably broader peak than native SWF, higher gelatinization onset temperature and peak gelatinization temperature, and lower enthalpy. This result suggests that native HAWF contains a lower proportion of double-helical structures than native SWF because the former has a lower amylopectin content, which requires less energy for dissociation [38]. In addition, peaks corresponding to amylose–lipid complexes were not detected in either native or HMT HAWF, possibly due to the broad gelatinization transition region overlapping with the amylose–lipid transition region.

HMT promotes the rearrangement of starch molecules, leading to the formation of more stable structures by increasing the double-helix content and facilitating the formation of amylose–lipid complexes, which are associated with the properties of RS [42]. Consequently, HMT inhibited starch gelatinization and enhanced thermal stability in both SWF and HAWF, as indicated by the increased gelatinization onset (T onset) and peak gelatinization (T peak) temperatures. This effect was more pronounced in HAWF than in SWF and was greater in HMT-25 than in HMT-20. The gelatinization peak of HAWF became noticeably narrower with increasing HMT moisture levels, possibly because of its higher amylose content, which promoted stronger amylose–amylose and amylose–amylopectin interactions [43]. Elevated gelatinization temperatures suggest that high-amylose content reinforces the molecular order within starch granules, requiring higher temperatures to disrupt these structures.

### 2.6. Crystallinity of Wheat Flours

**Figure 4:**
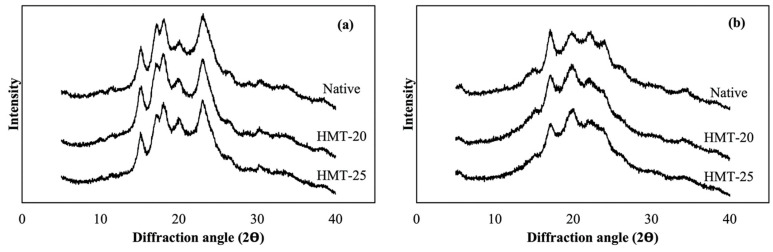
X-ray diffraction patterns of the wheat flour samples. (**a**) SWF, soft wheat flour; (**b**) HAWF, high-amylose wheat flour.

Figure 4 presents the X-ray diffraction patterns of the flour samples. HAWF exhibited a distinct B-type diffraction pattern, whereas SWF showed an A-type pattern, with diffraction peaks observed at 5.7°, 15.2°, 17.1°, 19.9°, 22.2°, and 23.9° for HAWF and at 15.2°, 17.1°, 18.1°, 20.1°, and 23.1° for SWF. These results indicate considerable differences in crystallinity. The crystalline patterns of SWF and HAWF after HMT changed in different ways. SWF exhibited slightly sharper peaks at 19.9°, whereas HAWF showed notable changes in the intensities of the peaks at 17.1° and 19.9°, along with the merging of the peaks at 22.2° and 23.9°. Brahma and Sit [44] reported that HMT-induced changes in the intensity of potato starch diffraction peaks were caused by the disruption of double helices within starch crystals, resulting in a more orderly structure than that of native starch.

### 2.7. SDS-Sedimentation Volume of Wheat Flours

**Figure 5:**
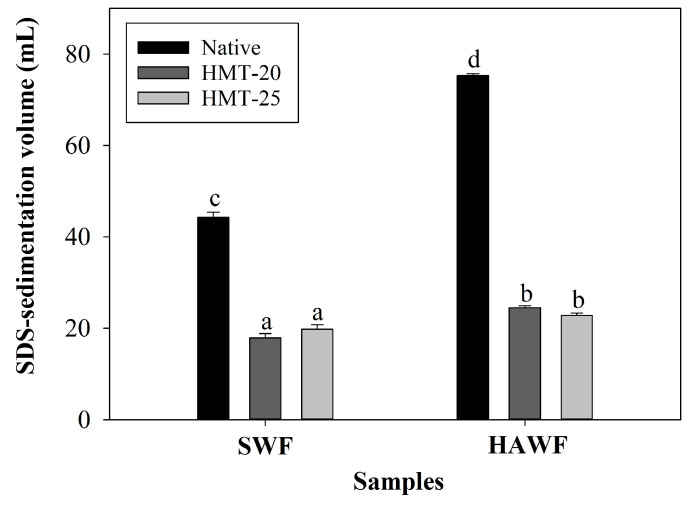
Sodium dodecyl sulfate (SDS)-sedimentation volume of the wheat flour samples. The different letters above the bars indicate significant differences (*p* < 0.05), according to Tukey’s HSD test.

Figure 5 presents the SDS-sedimentation volumes of the flour samples. SWF exhibited a lower SDS-sedimentation volume than HAWF, reflecting the weaker gluten strength of SWF. During cookie production, excessive gluten development negatively affects final product quality. The developed gluten hinders appropriate moisture evaporation from the dough, leading to reduced Maillard browning, inadequate color development, and poor cookie quality [27]. Therefore, HAWF, with its higher gluten strength, may produce smaller cookies than SWF, rendering it less suitable as a cookie flour. HMT significantly reduced the SDS-sedimentation volume, with a more pronounced effect in HAWF than in SWF. This reduction indicated weakened gluten strength due to protein denaturation during HMT, with greater denaturation observed in the higher-gluten HAWF. These results suggest that HMT enhances the cookie-making performance of flour. Although various methods—such as lactic acid SRC, SDS-sedimentation volume, and the wet gluten index—can be used to assess the gluten strength of flour, the SDS-sedimentation method has proven particularly useful for flours with varying particle sizes, such as whole wheat flour and wheat meals [45]. Therefore, the SDS-sedimentation volume results for native and HMT-treated flours with different particle sizes can sufficiently demonstrate the changes in gluten strength induced by HMT.

### 2.8. Dough-Mixing Property of Wheat Flours

**Figure 6:**
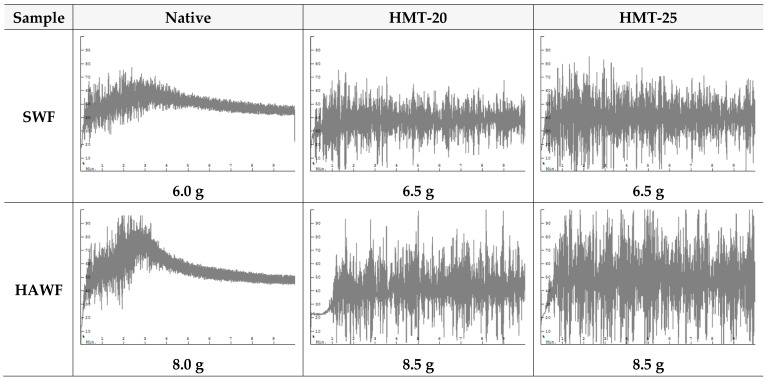
Mixograms of the wheat flour samples.

Figure 6 displays the dough properties of wheat flours. The dough mixing patterns of SWF samples were significantly different from those of HAWF samples. On the basis of the water SRC values, different water levels were used for mixing the dough (6.0–6.5 g for SWF and 8.0–8.5 g for HAWF). Native HAWF exhibited a considerably sharper peak pattern than native SWF. In contrast, the HMT samples of both SWF and HAWF did not show noticeable peaks in the dough-mixing patterns, possibly because of heat-induced gluten protein denaturation. Heat treatment causes gluten aggregation, resulting in decreased protein solubility and weakened dough strength owing to the lack of gluten network formation [31,32,46]. The dough appeared dry, as indicated by the highly fluctuating torque values recorded during pin mixing. For the HMT of both SWF and HAWF, dough mixing of HMT-25 flour exhibited greater fluctuation than that of HMT-20 flour when the same amount of water (6.5 g for SWF and 8.5 g for HAWF) was used. The results indicated that HAWF exhibited a higher water absorption than SWF, reflecting the SRC results. None of the mixed doughs developed gluten, which may be advantageous for cookie making, with a greater preference for dough prepared with HMT-25 flour.

### 2.9. Quality Characteristics of Cookies

**Figure 7:**
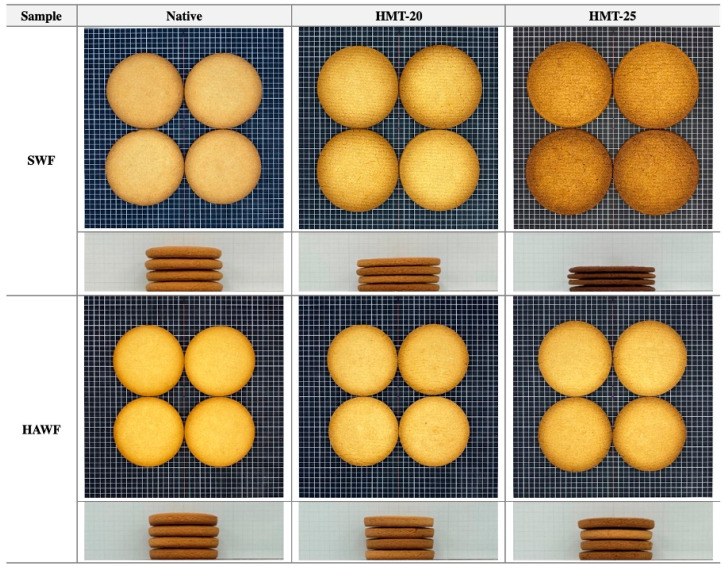
Top and side views of cookies formulated with wheat flour samples. Abbreviations: SWF, soft wheat flour; HAWF, high-amylose wheat flour; HMT, heat-moisture treatment.

Figure 7 displays the appearance of the cookies, and Table 5 presents their quality characteristics. Consistent with our expectations, based on the earlier results for SRC, pasting, thermal properties, SDS-sedimentation volume, and dough-mixing behavior, cookies prepared with SWF were larger than those prepared with HAWF. Cookies prepared with the HMT flour samples appeared larger in diameter and shorter in height, with a more pronounced effect observed for SWF. Notably, cookies prepared with SWF treated at 25% moisture appeared darker than the other samples. In terms of cookie dimensions, HMT demonstrated benefits in desirable cookie attributes, which typically include lower moisture content (due to greater moisture loss during baking), larger cookie diameter, and thinner height. The color development in cookies is primarily driven by heat-induced mechanisms, including the Maillard reaction and caramelization. Browning progresses through distinct phases during baking. Initially, moisture loss leads to increased lightness. As the temperature of the cookie rises, a rapid browning phase occurs due to the formation of melanoidins via the Maillard reaction. Towards the end of baking, darker tones develop as a result of continued Maillard reactions and/or caramelization, particularly in low-moisture areas such as the cookie bottom [47].

As reflected in the cookie appearance, the L* and b* values of cookies prepared with native SWF were lower than those of cookies prepared with native HAWF; HMT flour samples treated at 25% moisture showed a significant decrease in both L* and b* values (*p* < 0.05); the effect was more pronounced in HAWF (Table 5). The results confirmed a negative relationship between L* and cookie diameter [27,48]. The darker, more golden-brown color observed in the HMT cookies may be attributed to structural changes caused by heat transfer to the dough and enhanced non-enzymatic browning reactions.

Moisture loss during baking was higher for the cookies prepared with native SWF (14.9%) than for those prepared with native HAWF (14.3%). Cookies prepared with HMT flours exhibited increased moisture loss (15–16.4% for SWF and 14.5–14.6% for HAWF), possibly because the dough matrix was weakened by gluten denaturation, which facilitated moisture evaporation [44]. Accelerated surface dehydration during baking increases cookie dough temperature, leading to the Maillard reaction and generation of melanoidins, which contribute to a dark and brown color.

The cookie diameter was significantly larger for cookies prepared with native SWF (74.9 mm) than for cookies prepared with native HAWF (66.9 mm) (*p* < 0.05). The HMT of flour further increased cookie diameter, with a greater extent of increase observed with SWF (78.9–82.4 mm) than with HAWF (66.3–70.3 mm). In contrast, cookie height was significantly lower for cookies prepared with native SWF (40.3 mm) than for those prepared with native HAWF (44.7 mm) (*p* < 0.05). The HMT of flour significantly reduced the height of cookies prepared with SWF and slightly reduced the height of cookies prepared with HAWF. Additionally, a strong negative correlation was found between cookie diameter and height.

The spread ratio, calculated as the diameter divided by height, was significantly higher for cookies prepared with native SWF than for those prepared with native HAWF (*p* < 0.05). Additionally, the spread ratio of cookies prepared with HMT flour increased significantly, with the effect being more pronounced with SWF.

Correlation analysis between flour quality and cookie quality revealed significant relationships involving amylose content, particle size, solvent retention capacity (SRC), and SDS-sedimentation volume. Amylose content was significantly correlated with cookie diameter (r = −0.92**), height (r = 0.74**), and spread ratio (r = −0.72**). Particle size parameters (d50, d75, and d90) also showed significant correlations, with d75 being particularly notable: r = 0.62**, −0.79**, and 0.79** for cookie diameter, height, and spread ratio, respectively. SRC in lactic acid, sodium carbonate, and sucrose solutions exhibited significant correlations, especially lactic acid SRC, with r = −0.84**, 0.86**, and −0.81** for cookie diameter, height, and spread ratio, respectively (**, *p* < 0.01). Additionally, SDS-sedimentation volume, particularly SDS-60, showed significant correlations with r = −0.46*, 0.54*, and −0.46* for cookie diameter, height, and spread ratio, respectively (*, *p* < 0.05).

In summary, cookies prepared with the HMT flour showed a significant improvement in quality, including reduced moisture content, increased diameter, and decreased height. These outcomes were primarily attributed to the HMT flour exhibiting more ordered starch granules, denatured gluten protein, densified flour particles, and increased flour particle size compared with the native flour. These factors collectively contribute to the final cookie quality. In our study, the particle sizes of the HMT flour were significantly larger than those of the native flour; thus, cookie characteristics may be influenced by the particle size of the flour, in addition to the effects of HMT on flour components such as starch and gluten protein. Therefore, it may be beneficial to investigate the cookie-making performance of HMT flour with particle sizes adjusted to match those of native flour by further grinding, which would allow a more precise assessment of the effects of HMT on flour components.

### 2.10. In Vitro Digestibility of Defatted Cookie Samples

Table 6 presents the in vitro digestibility of the defatted cookie samples. Cookies prepared with native SWF contained 40.9% rapidly digestible starch (RDS), 8.4% SDS, and 0.3% RS. In contrast, cookies prepared with native HAWF exhibited 25.3% RDS, 9.6% SDS, and 7.2% RS. The results indicated significantly lower digestibility for HAWF than for SWF, which may be attributed to differences in amylose content, as well as in the distribution of amylopectin branch chain length in the starch of each flour. Chung et al. [49] and Kweon et al. [50] have reported that RS content exhibited a positive correlation with both amylose content and amylopectin branch length and a negative correlation with the molecular weight of amylose in rice starch. Li et al. [51] reported higher RDS content in noodles prepared from high-amylose flour than in noodles prepared from normal wheat flour.

For cookies prepared with HMT flour, both SWF and HAWF exhibited a significant decrease in RDS (37.1–39.4% for SWF and 23.1–23.6% for HAWF) and a significant increase in SDS (9.9–11.5% for SWF and 10.5–10.7% for HAWF). Additionally, RS content showed no significant change in SWF (remaining 0.3%) but increased significantly in HAWF (7.6–8.5%). These findings suggest that HMT induces structural modifications that delay starch digestion, possibly due to increased starch crystallinity rather than amylose–lipid complex formation (which was not observed in the present study). The ratio of RS to total starch (TS) content was significantly higher with HAWF (16.8%) than with SWF (0.6%). Furthermore, HMT increased the proportion of RS in TS to 17.5% and 19.2% for HAWF and SWF, respectively. Previous studies on the heat-moisture treatment (HMT) of high-amylose corn and rice starches have shown a considerable increase in resistant starch (RS) content, although the extent of the increase depends on treatment conditions such as moisture level, temperature, and duration [52,53].

Based on correlation analysis, significant relationships between flour quality and cookie digestibility were observed for amylose content, particle size, solvent retention capacity (SRC), and pasting properties. Amylose content showed strong correlations with rapidly digestible starch (RDS), total digestible starch (TDS), and resistant starch (RS), with correlation coefficients of r = −0.98**, −0.99**, and 0.99**, respectively. Particle size parameters (d10, d25, d50, d75, and d90) were significantly correlated with slowly digestible starch (SDS), with r = 0.76**, 0.88**, 0.88**, 0.83**, and 0.85**, respectively. SRC in water, lactic acid, sodium carbonate, and sucrose also showed significant correlations with RDS, TDS, and RS. In particular, sodium carbonate and sucrose SRC values exhibited strong correlations: for RDS, r = −0.95** and 0.95**; for TDS, r = −0.95** and 0.94**; and for RS, r = 0.93** and 0.97**, respectively (*p* < 0.01). Pasting properties—including peak, breakdown, final, and setback viscosities—were also significantly correlated with digestibility, with r = 0.92–0.95** for RDS, r = 0.96–0.97** for TDS, and r = −0.96 to −0.97** for RS (*p* < 0.01).

In summary, HMT is an effective method for controlling starch digestibility, particularly by enhancing slow digestion and RS formation in cookies prepared with HAWF, which suggests its potential applications for the development of functional foods.

The present study hypothesized that applying HMT to HAWF would induce the denaturation of gluten proteins, thereby improving the baking performance of the flour and enhancing the appearance of the final product. Collectively, the results supported this hypothesis; however, the increase in diameter was less pronounced than expected, particularly compared with the increase observed with SWF. In addition, HMT of HAWF provided nutritional benefits by reducing digestibility in cookies prepared with HMT flour. Thus, HAWF proved to be an excellent ingredient for producing cookies with enhanced prebiotic effects via HMT. However, the present study was limited by the HMT conditions applied. Further in-depth investigations to optimize HMT conditions would be useful for maximizing both functional and nutritional benefits.

## 3. Materials and Methods

### 3.1. Materials

HAWF was provided by Arista Cereal Technologies Pty Ltd. (Sydney, Australia), which was milled from high-amylose wheat varieties bred using traditional breeding methods without genetic modification (non-GMO). Commercial SWF (Samyang, Seoul, Republic of Korea) was used as the control. For cookie making, shortening (Silver Shortening-Free; Lotte Wellfood Co., Seoul, Republic of Korea), consisting of a blend of palm oil and esterified palm oil, was purchased from a local market.

### 3.2. HMT of Wheat Flours

An appropriate amount of distilled water was added to 500 g of wheat flour, calculated on the basis of its moisture content. The mixture was homogenized using a grinder. The uniformly mixed samples were placed in a stainless-steel container (Ø203 × 50 mm), sealed with a stainless-steel cover and aluminum foil, and heated at 130 °C for 1 h in a dry oven. The HMT conditions used in this study (130 °C for 1 h) were selected based on preliminary experiments that evaluated a range of moisture levels (15–30%), temperatures (110–140 °C), and treatment durations (30–180 min). Among these, HMT at 130 °C for 1 h consistently produced the most favorable outcomes, including increased resistant starch content and reduced starch digestibility. After HMT, the samples were cooled to 20–25 °C for 24 h and subsequently passed through a 500 mesh sieve (500 μm opening).

### 3.3. Particle Size Analysis of Wheat Flours

The particle sizes and distribution of the HMT flour samples were measured using a particle size analyzer (LS 13 320, Beckman Coulter, Brea, CA, USA), as per the dry method.

### 3.4. Analysis of Moisture, Amylose, and Damaged Starch Content of Wheat Flours

The moisture content of wheat flour samples was measured according to the American Association of Cereal Chemists (AACC) Method 44-15.02 [54]. The amylose content was determined using the iodine–starch reaction. The sample (20 mg) was mixed with 2 mL of 1N sodium hydroxide and 4 mL of distilled water and boiled for 30 min. A 0.25 mL aliquot from the resulting solution was added to 25 mL of trichloroacetic acid (0.5%), followed by the addition of 0.25 mL of iodine solution (2.0 g KI + 0.2 g I_2_ in 100 mL of distilled water). After 30 min, the absorbance was measured at 620 nm. Amylose (from potatoes; Sigma-Aldrich, St. Louis, MO, USA) and amylopectin (from maize; Sigma-Aldrich) were used to prepare a standard curve to calculate the amylose content of the sample. Starch damage content was determined using a starch damage assay kit (K-SDAM; Megazyme Ltd., Wicklow, Ireland).

### 3.5. Analysis of SRC of Wheat Flours

The SRC of the HMT flour samples in distilled water, 5% lactic acid, 5% sodium carbonate, and 50% (*w*/*w*) sucrose solution were measured using the AACC Method 56-11.02 [54].

### 3.6. Analysis of Pasting Properties of Wheat Flours

The pasting properties of the HMT flour samples were measured using an RVA 4 (Newport Scientific, Sydney, Australia). The flour (3.5 g) and distilled water (25 mL) were placed in an RVA canister and mixed thoroughly using a plastic paddle to prevent lump formation. The RVA was then operated for 13 min using the Standard 1 method. Pasting parameters were calculated using Thermocline for Windows, version 2.5 (Newport Scientific).

### 3.7. Analysis of Thermal Properties of Wheat Flours

The thermal characteristics of starch in the HMT flour samples were measured using DSC. Flour and distilled water were mixed in a 1:1 (*w*/*w*) ratio, and approximately 40 mg of the mixture was placed in an empty aluminum pan and sealed. The sealed pan was placed in a DSC instrument (DSC 6000, PerkinElmer Co., Waltham, MA, USA) and heated from 10 °C to 135 °C at a rate of 10 °C/min. An empty aluminum pan was used as the reference. Gelatinization temperatures and enthalpies were calculated using the Pyris Software (version 11, PerkinElmer, Waltham, MA, USA).

### 3.8. Analysis of Crystallinity of Wheat Flours

The crystalline patterns of starch granules in the HMT flour samples were measured using an X-ray diffractometer (X’Pert; Malvern Panalytical, Malvern, UK). The measurement was conducted under the following conditions: target, Cu-Kα; scanning speed, 4°/min; voltage, 40 kV; current, 30 mV; and diffraction angle (2θ), 5 to 40°.

### 3.9. Measurement of SDS-Sedimentation Volume of Wheat Flours

SDS-sedimentation tests were performed according to the AACC Method 56-70.01 [54] to assess the relative gluten strength in wheat flour. The wheat flour sample (5 g) was placed in a 100 mL graduated cylinder, and 50 mL of distilled water was added. The cylinder was closed and shaken vertically and horizontally to hydrate the samples. Subsequently, 50 mL of 3% SDS–lactic acid solution was added, and the mixture was shaken again. The cylinder was subsequently set upright, and the volume (mL) of the sedimented sample was precisely recorded at 40 min.

### 3.10. Measurement of Mixed Dough Property of Wheat Flours

The dough properties of the wheat flour samples were measured using a 10 g mixograph (National Manufacturing Co., Lincoln, NE, USA) following the AACC Method 54-40.02 [54]. The flour sample (10 g) was placed in the mixing bowl, and distilled water, 6.0–6.5 g of soft wheat, and 8.0–8.5 g of high-amylose wheat were added. The mixture was kneaded for 10 min, and the mixing curve was recorded to analyze dough properties.

### 3.11. Preparation of Cookies with Wheat Flours

Cookie samples were prepared according to the AACC Method 10-53.01 [54]. Shortening (90 g) and sucrose (94.5 g) were weighed separately, placed in a mixing bowl (N50; Hobart, Troy, OH, USA), and mixed for 3 min at speed 1 (low speed). Syrup (3.4 g), water (49.5 g), and ammonia bicarbonate (1.1 g) were combined, added to the bowl, and mixed at speed 1 for 1 min, followed by mixing at speed 2 (medium speed) for an additional 1 min. Skim milk powder (2.3 g), salt (2.8 g), and sodium bicarbonate (2.3 g) were thoroughly mixed with flour (225 g); added to the bowl; and mixed for 2 min at speed 1. After mixing, the dough was divided into four portions of 60 g each and placed on an aluminum cookie sheet (25.4 × 33.0 cm) with two metal gauge strips (7 mm thick) attached along the long edges of the sheet. Each dough portion was flattened using a rolling pin and shaped using a circular cutter (6 cm diameter).

### 3.12. Analysis of Quality of Cookies

Cookie dimensions (length and width) were measured individually for four cookies, and the average values were calculated. Cookie height was measured using a caliper by vertically stacking four cookies, and the average height was calculated from four measurements obtained in different stacking orders. The top surface color of the cookies was measured using a colorimeter (CR-20; Minolta Co., Tokyo, Japan) on the basis of L* (lightness), a* (redness), and b* (yellowness) values. Measurements were taken four times, and the average was calculated.

### 3.13. Analysis of In Vitro Digestibility of Wheat Flours and Cookies

The digestibility of the wheat flour and cookies was determined by measuring the content of RDS, SDS, total digestible starch, and RS, following the manufacturer’s protocol, using a digestible starch assay kit (K-DSTRS; Megazyme Ltd., Wicklow, Ireland). Prior to analysis, the cookies were ground and defatted with petroleum ether at a 1:5 (*w*/*w*) ratio by stirring at a low speed (140 rpm) with a magnetic stirrer for 24 h.

### 3.14. Statistical Analysis

All data were obtained from measurements repeated at least thrice. Differences between sample means were analyzed by analysis of variance followed by Tukey’s HSD test at *p* < 0.05, using the SPSS 29.0 software (SPSS Inc., Armonk, NY, USA).

## 4. Conclusions

The present study demonstrates that HMT at moisture levels of 20–25% and a temperature of 130 °C for 1 h significantly alters the physicochemical and functional properties of HAWF and SWF, thereby enhancing their suitability for cookie production. HMT increases flour particle size and crystallinity, reduces damaged starch content, and modifies pasting and thermal behavior. These changes were attributed to flour particle agglomeration, increased starch reordering, and protein denaturation, leading to altered dough rheology. Consequently, cookies prepared from HMT flours exhibited larger diameters, reduced heights, and higher spread ratios, which are associated with a desirable texture, than cookies prepared from native flour; these effects were more pronounced with SWF than with HAWF. Additionally, HMT increased the resistant starch (RS) content by approximately 20%, particularly in HAWF-based cookies, suggesting potential health benefits. Overall, the effects of HMT—particularly the influence of moisture level on the quality and nutritional characteristics of both flour and cookies—were more pronounced at the higher moisture level (25%) than at the lower moisture level (20%). In summary, HMT is emerging as a promising physical modification technique for improving cookie quality and nutritional profile. Future research should focus on optimizing HMT conditions to balance functional improvements with sensory qualities.

## Figures and Tables

**Table 1:** Particle size of the wheat flour samples.

Sample		d10	d25	d50	d75	d90
SWF	Native	7.9 ± 0.4 ^a^	15.3 ± 0.4 ^a^	28.1 ± 0.7 ^a^	70.5 ± 1.0 ^a^	103.8 ± 0.0 ^a^
	HMT-20	18.5 ± 0.4 ^b^	33.3 ± 0.7 ^b^	83.4 ± 1.0 ^b^	201.3 ± 4.3 ^b^	381.8 ± 3.2 ^d^
	HMT-25	46.0 ± 1.5 ^d^	96.0 ± 1.4 ^f^	236.3 ± 4.7 ^e^	426.1 ± 3.0 ^c^	568.9 ± 2.8 ^f^
HAWF	Native	18.8 ± 0.1 ^b^	44.4 ± 0.3 ^c^	78.7 ± 0.1 ^b^	114.2 ± 0.3 ^d^	148.1 ± 1.2 ^b^
	HMT-20	39.7 ± 0.4 ^c^	65.5 ± 0.4 ^d^	100.5 ± 0.5 ^c^	158.6 ± 0.7 ^e^	321.2 ± 3.8 ^c^
	HMT-25	60.5 ± 1.2 ^e^	88.9 ± 1.3 ^e^	141.8 ± 2.4 ^d^	287.1 ± 7.7 ^f^	470.5 ± 2.9 ^e^

Note: Mean values (±standard deviation) (*n* = 3) with different letters within the same column are significantly different (*p* < 0.05), according to Tukey’s HSD test. Abbreviations: SWF, soft wheat flour; HAWF, high-amylose wheat flour; HMT, heat-moisture treatment; HMT-20, heat-moisture treatment at 20% moisture levels; HMT-25, heat-moisture treatment at 25% moisture levels. The parameters d10, d25, d50, d75, and d90 denote the particle diameters corresponding to the 10th, 25th, 50th, 75th, and 90th percentiles of the cumulative volume distribution, respectively.

**Table 2:** Moisture, amylose, and damaged starch contents and solvent retention capacity of the wheat flour samples.

Sample		Moisture (%)	Amylose (%, dwb)	Starch Damage (%, dwb)	Solvent Retention Capacity (%)			
					Water	Lactic Acid	Sodium Carbonate	Sucrose
SWF	Native	13.6 ± 0.0 ^e^	21.4 ± 0.2 ^b^	3.4 ± 0.0 ^e^	51.2 ± 0.3 ^a^	103.1 ± 1.1 ^bc^	72.0 ± 0.0 ^b^	96.4 ± 1.1 ^c^
	HMT-20	10.0 ± 0.0 ^a^	20.6 ± 0.3 ^a^	2.0 ± 0.0 ^c^	65.7 ± 0.3 ^b^	89.3 ± 1.1 ^a^	68.9 ± 0.1 ^a^	88.2 ± 0.3 ^a^
	HMT-25	10.8 ± 0.0 ^c^	21.8 ± 0.4 ^b^	2.8 ± 0.0 ^d^	68.0 ± 0.1 ^c^	89.0 ± 0.3 ^a^	73.7 ± 0.1 ^c^	91.4 ± 0.8 ^b^
HAWF	Native	15.2 ± 0.0 ^f^	46.7 ± 0.1 ^d^	4.7 ± 0.0 ^f^	75.5 ± 0.3 ^d^	118.9 ± 0.4 ^d^	89.6 ± 0.0 ^e^	128.5 ± 1.5 ^d^
	HMT-20	10.2 ± 0.0 ^b^	47.0 ± 0.2 ^d^	1.5 ± 0.0 ^a^	99.0 ± 0.6 ^e^	101.6 ± 0.1 ^b^	97.7 ± 0.5 ^f^	155.3 ± 0.6 ^e^
	HMT-25	11.6 ± 0.0 ^d^	44.5 ± 0.4 ^c^	1.6 ± 0.0 ^b^	99.9 ± 0.4 ^e^	104.7 ± 0.2 ^c^	88.1 ± 0.0 ^d^	159.3 ± 1.2 ^f^

Note: Mean values (±standard deviation) (*n* = 3) with different letters within the same column are significantly different (*p* < 0.05), according to Tukey’s HSD test. Abbreviations: SWF, soft wheat flour; HAWF, high-amylose wheat flour; HMT, heat-moisture treatment; dwb, dry weight basis.

**Table 3:** Pasting properties of wheat flour samples.

Sample		Peak Viscosity (cP)	Final Viscosity (cP)	Setback Viscosity (cP)	Pasting Temperature (°C)
SWF	Native	2755 ± 8 ^c^	3294 ± 9 ^c^	1342 ± 23 ^b^	64.5 ± 1.1 ^a^
	HMT-20	4241 ± 45 ^e^	4938 ± 35 ^d^	1894 ± 1 ^c^	69.0 ± 0.6 ^b^
	HMT-25	3803 ± 16 ^d^	5027 ± 15 ^e^	2040 ± 54 ^d^	71.0 ± 0.1 ^b^
HAWF	Native	368 ± 5 ^b^	361 ± 1 ^b^	16 ± 1 ^a^	81.2 ± 0.7 ^c^
	HMT-20	313 ± 5 ^ab^	306 ± 8 ^ab^	12 ± 2 ^a^	81.9 ± 1.7 ^c^
	HMT-25	283 ± 0 ^a^	277 ± 1 ^a^	7 ± 0 ^a^	82.3 ± 1.1 ^c^

Note: Mean values (±standard deviation) (*n* = 2) with different letters within the same column are significantly different (*p* < 0.05), according to Tukey’s HSD test. Abbreviations: SWF, soft wheat flour; HAWF, high-amylose wheat flour; HMT, heat-moisture treatment.

**Table 4:** Thermal properties of wheat flour samples.

		Gelatinization Peak			Amylose–Lipid Complex Peak		
		T Onset (°C)	T Peak (°C)	ΔH (J/g)	T Onset (°C)	T Peak (°C)	ΔH (J/g)
SWF	Native	61.8 ± 0.1 ^b^	68.4 ± 0.1 ^a^	3.74 ± 0.15 ^a^	97.5 ± 0.6 ^b^	108.0 ± 0.6 ^a^	0.41 ± 0.04 ^a^
	HMT-20	63.5 ± 0.1 ^c^	70.9 ± 0.1 ^b^	3.72 ± 0.08 ^a^	95.3 ± 0.4 ^a^	109.2 ± 0.3 ^a^	0.44 ± 0.00 ^a^
	HMT-25	65.3 ± 0.2 ^d^	76.7 ± 0.0 ^c^	3.35 ± 0.00 ^a^	98.8 ± 0.6 ^b^	109.1 ± 0.0 ^a^	0.40 ± 0.01 ^a^
HAWF	Native	60.6 ± 0.1 ^a^	82.9 ± 0.6 ^d^	3.54 ± 0.17 ^a^	nd	nd	nd
	HMT-20	70.8 ± 0.3 ^e^	89.5 ± 0.5 ^e^	3.58 ± 0.09 ^a^	nd	nd	nd
	HMT-25	77.5 ± 0.7 ^f^	94.3 ± 0.5 ^f^	3.52 ± 0.02 ^a^	nd	nd	nd

Note: Mean values (±standard deviation) (*n* = 3) with different letters within the same column are significantly different (*p* < 0.05), according to Tukey’s HSD test. Nd, not detected. Abbreviations: SWF, soft wheat flour; HAWF, high-amylose wheat flour; HMT, heat-moisture treatment; T peak, peak gelatinization temperature; T onset, gelatinization onset temperature; ΔH, enthalpy.

**Table 5:** Quality characteristics of cookies formulated with wheat flour samples.

Sample		Moisture Loss (%)	Top Surface Color			Diameter (mm)	Height (mm)	Spread Ratio (Dia./Height)
			L*	a*	b*			
SWF	Native	14.7 ± 0.0 ^c^	62.3 ± 1.6 ^b^	14.4 ± 0.7 ^a^	33.0 ± 0.4 ^bc^	74.9 ± 0.0 ^d^	40.3 ± 0.1 ^c^	7.4 ± 0.0 ^d^
	HMT-20	17.1 ± 0.0 ^d^	61.4 ± 1.4 ^b^	13.7 ± 0.7 ^a^	31.9 ± 0.6 ^b^	78.9 ± 0.1 ^e^	32.4 ± 0.1 ^b^	9.7 ± 0.0 ^e^
	HMT-25	17.5 ± 0.0 ^e^	38.2 ± 4.4 ^a^	13.8 ± 0.5 ^a^	22.9 ± 2.5 ^a^	82.4 ± 0.0 ^f^	24.0 ± 0.1 ^a^	13.7 ± 0.0 ^f^
HAWF	Native	14.2 ± 0.0 ^a^	65.0 ± 1.1 ^c^	14.1 ± 0.5 ^a^	34.8 ± 0.5 ^d^	66.9 ± 0.0 ^b^	44.7 ± 0.1 ^f^	6.0 ± 0.0 ^a^
	HMT-20	14.6 ± 0.0 ^b^	64.9 ± 1.2 ^c^	14.3 ± 0.5 ^a^	33.7 ± 0.5 ^cd^	66.3 ± 0.0 ^a^	42.4 ± 0.1 ^e^	6.3 ± 0.0 ^b^
	HMT-25	14.6 ± 0.0 ^b^	61.7 ± 2.3 ^b^	15.2 ± 0.9 ^b^	33.5 ± 0.3 ^c^	70.3 ± 0.0 ^c^	41.0 ± 0.2 ^d^	6.9 ± 0.0 ^c^

Note: Mean values (±standard deviation) (*n* = 4) with different letters within the same column are significantly different (*p* < 0.05), according to Tukey’s HSD test. Abbreviations: SWF, soft wheat flour; HAWF, high-amylose wheat flour; HMT, heat-moisture treatment; Dia., diameter.

**Table 6:** RDS, SDS, TDS, and RS content of defatted cookie samples (%, as is).

		RDS (%)	SDS (%)	TDS (%)	RS (%)
SWF	Native	40.9 ± 0.0 ^e^	8.4 ± 0.2 ^a^	48.7 ± 0.2 ^b^	0.3 ± 0.0 ^a^
	HMT-20	39.4 ± 0.2 ^d^	9.9 ± 0.6 ^b^	48.9 ± 0.1 ^b^	0.3 ± 0.0 ^a^
	HMT-25	37.1 ± 0.4 ^c^	11.5 ± 0.3 ^c^	48.9 ± 0.1 ^b^	0.3 ± 0.0 ^a^
HAWF	Native	25.3 ± 0.0 ^b^	9.6 ± 0.2 ^ab^	35.7 ± 0.0 ^a^	7.2 ± 0.0 ^b^
	HMT-20	23.6 ± 0.2 ^a^	10.7 ± 0.3 ^bc^	35.6 ± 0.2 ^a^	7.6 ± 0.1 ^c^
	HMT-25	23.1 ± 0.1 ^a^	10.5 ± 0.0 ^bc^	35.8 ± 0.3 ^a^	8.5 ± 0.0 ^d^

Note: Mean values (± standard deviation) (*n* = 3) with different letters within the same column are significantly different (*p* < 0.05), according to Tukey’s HSD test. Abbreviations: SWF, soft wheat flour; HAWF, high-amylose wheat flour; HMT, heat-moisture treatment; RDS, rapidly digestible starch; SDS, slowly digestible starch; TDS, total digestible starch; RS, resistant starch.

## Data Availability

The data presented in this study are available upon request from the corresponding author.

## Abbreviations

The following abbreviations are used in this manuscript:

HMT	Heat-moisture treatment
SRC	Solvent-retention capacity
SDS	Sodium dodecyl sulfate
RVA	Rapid viscosity analyzer
DSC	Differential scanning calorimetry
RS	Resistant starch
TS	Total starch
RDS	Rapidly digestible starch
TDS	Total digestible starch
